# On Three Cases of Deformity Resulting from Hip-Joint Disease

**Published:** 1898-04-02

**Authors:** Alfred Willett

**Affiliations:** Surgeon to the Hospital


### ON THREE CASES OF DEFORMITY RESULTING FROM HIP-JOINT DISEASE.

Abstract of a Clinical Lecture given at St. Bartholomew's Hospital by Alfred Willett, F.R.C.S., Surgeon to the Hospital, on November 3rd, 1897.

The first case, a female, aged 13, was admitted to the above hospital on October 21st, 1897. The history was that when three years of age she began to get lame, but that the hip-joints were movable until the age of nine years, after which the hips became anchylosed. The family history was negative. Condition on admission: Both hip-joints are anchylosed, and there is great lumbar lordosis. The left thigh is flexed and adducted; the right thigh simply flexed. When the trunk is straight in bed, the right thigh makes an angle of 130 deg. with the body, the left thigh an angle of 120 deg. The measurement from the anterior superior iliac spine to the outer side of the knee-joint is, on the left side, $13\frac{3}{4}$ in.; on the right side, 14 in. The left thigh is more adducted and internally rotated than the right thigh. There are no enlarged glands in the groin.

The patient was introduced and put on a couch, and it was pointed out that, when fully extended, marked lumbar lordosis was present, and the correct method of accurately estimating deformity in anchylosed hip-joint was demonstrated. Mr. Willett then grasped the patient's thigh, and moved each thigh one after the other. He pointed out that this movement caused no pain, and that the pelvis moved in all directions in correspondence with movements of the thighs, as though thighs and pelvis were all firmly blended. The patient was then asked to walk, and it was pointed out that she walked with the knees flexed and kept always close together, causing her to assume an odd, shuffling gait.

Mr. Willett next discussed the etiology of this case, and he remarked that disease of the hip-joints in children is always, or nearly always, tubercular, and that to have both limbs affected occurs in only 1 or 2 per cent. of all cases of hip-joint disease of this type. This anchylosis has occurred without suppuration, and it probably commenced as a tubercular synovitis proper —that is to say that the disease was at first limited to the synovial membrane. I say this—Mr. Willett remarked—because from the history we see that the affection was all along of a chronic type, and that she had never had any very characteristic symptoms of hip-joint disease; the disease nevertheless had its termination in anchylosis.

The question of treatment was next discussed, and it was remarked that she could never be fit for any occupation other than sedentary, owing to her being so severely crippled; for whilst when one hip only is affected the other hip-joint in a measure is capable of doing duty for both, when both are anchlyosed the patient is reduced to a shuffling progression, as in this patient —that is, she paddles from below her knees. As to operative treatment, the femora might be divided below the trochanters, but the manner of progression would not be improved thereby. So it is intended to excise the neck and part of the great trochanter on the right side by means of an anterior incision, following the lines of excision of a carious head of the femur. After excision for hip-joint disease has been performed bony union does not occur, but a good fibrous union takes place, which should always result if sufficient bone is removed. Moreover, if, as is intended, the neck with a portion of the greater trochanter of the right femur are removed, and the limb kept on an extension apparatus, recovery with a movable false joint may be anticipated. Adams' osteotomy of the left limb will also be performed. In the result both legs should be of equal length, and with a movable hip-joint on the right side.*

The next case was a lad, aged 17, who was admitted on October 21st, 1897. The history was that in Christmas, 1895, the patient is said to have had rheumatic fever, which kept him in bed for four months, and that during that period only the right hip was affected, and also that during this period his right foot became turned outwards, and his right leg became stiff in the extended position. There was pain in the hip and knee. At the end of the four months he got up, and walked by the aid of two sticks until June, 1896. He was admitted into St. Bartholomew's Hospital in July, 1896, and was put upon an extension apparatus and a "Thomas's splint" for three months. Since then he has never walked properly.

The comparative measurements, &c., are as follows: The right leg is everted, and $3\frac{1}{2}$ in. shorter than the left. Flexion and extension take place in the coronal plain. The great trochanter is 1 in. above Nelaton's line. The great trochanter is only 1 in. below a horizontal line drawn through the two anterior superior iliac spines, but the left trochanter is $3\frac{3}{4}$ in. below this line. The right trochanter is very enlarged and prominent. The measurement from the internal tuberosity of the right tibia to its internal malleolus is $14\frac{3}{4}$ in.; and from the inner tuberosity of the left tibia to its internal malleolus is $15\frac{1}{2}$ in. All movements at the right hip are abrogated. There are scars of old abscesses at the back of the right calf.

These features were demonstrated by Mr. Willett, who showed that the top of the great trochanter was almost on the same level as a horizontal line drawn through the two anterior superior iliac spines, and that the right leg was rotated outwards. The right leg was moved in every direction, the movement causing no pain because the hip-joint was anchylosed. It was shown that by

* The operations as described were shortly afterwards performed with satisfactory results.

using the fingers as calipers with which to compare both trochanters, great over-production of bone in front of the right great trochanter could be defined. This case showed well the secondary results of anchylosis, namely, great muscular wasting in the right leg and atrophy, for the right tibia had failed to grow to the size of the left one.

The points which were next considered were: (1) The nature of the original disease; (2) how the deformity arose; (3) the present condition of the limb: (4) the treatment to be adopted.

(1) The nature of the original disease: It was an acute tuberculosis of the hip-joint. It was not acute rheumatism, because the history shows that the right hip only was affected, and he has gone through many of the phases of ordinary tubercular hip-joint disease. (2) How the deformity arose: Probably the stress of the disease was on the pelvic side of the articulation—that is to say, in the acetabulum; for we have in this case to reconcile shortening, eversion, and thickening, with a prominent trochanter. It might be supposed that he had an early dislocation of the hip-joint, but in dislocation there would not be so much outgrowth of bone nor loss of motion. It might also be suggested that the disease began at the epiphiseal line; but in such a case the head of the bone dies and there is suppuration, and the trochanter would probably be sunken instead of prominent as it is here. Lastly, and the probability is, that the stress of the disease was on the pelvic side of the articulation. This would explain the shortening and prominence of the trochanter, for in such a case the head of the femur would be pushed upwards and outwards from the acetabulum; there is no *à priori* reason why the acetabulum should not be affected as well as the head of the femur. A skiograph was taken of this man's pelvis and thigh, but it merely shows the prominence of the trochanter—everything else being blurred. (3) The present condition of the limb: There is presumedly erosion of and enlargement of the area of the acetabulum, producing shortening; and a large quantity of new bone has been formed. (4) The treatment to be adopted: It is proposed to do a Gant's osteomoty of the femur—*i.e.*, below the trochanters—immediately.*

The next case was a man aged 64, who was admitted on August 19th, 1897. The history was that 26 years ago a basket of coal fell on to his left hip, which gave him little trouble at the time. But six months later he had pain in the left hip, which kept him in bed for three months. Six months still later abscesses appeared first in the left hip, then in the right hip. They were opened, and healed, after which he walked well. Fourteen years ago both hips began to get stiff, and have since then gradually become stiffer and more adducted. Family history: There is a strong tuberculous history. On admission the right thigh is adducted, so that the knee is 4 in. beyond the middle line, and flexed 10 deg. There is a very small amount of movement at hip-joint. The left thigh is similarly adducted, but not flexed. It is firmly anchylosed. There is no dislocation at either hip, and no pain. On September 20th a 1-in. incision was made from the lower part of the left great trochanter downwards. A groove was then made in the bone with an Adams' saw, a chisel was then inserted into this groove, and the bone divided. The limb was then put in good position. The right femur was then treated in the same way. A Thomas's splint and a 10 lb. extension were then applied to each leg. On October 6th the extension apparatus was taken off. On October 22nd the Thomas's splints were taken off.

* This operation was performed with very satisfactory results.

Mr. Willett remarked that this man walked by means of what has been described as "cross-legged or scissor progression," *i.e.*, the legs were crossed and presented the appearance of a pair of scissors. The original disease was either rheumatism, osteo-arthritis, or tubercle; but his family history, and the fact that he had abscesses, make it probable that it was tubercle. He was admitted to the above hospital because it was thought that his legs could be put straight, and his progression thereby improved, for he has developed Dupuytrous contraction of palmar fusion in both hands, and cannot fully grasp a stick.

